# In the Search for the Treatment of Compensatory Sweating

**DOI:** 10.1100/2012/134547

**Published:** 2012-09-17

**Authors:** Tomasz Stefaniak, Marta Cwigon, Dariusz Łaski

**Affiliations:** ^1^Department of General, Endocrine and Transplant Surgery, Medical University of Gdansk, 7 Debinki Street, PL-80-210 Gdansk, Poland; ^2^Pomeranian Foundation for Progress in Surgery, 44 Wilenska Street, PL-80-215 Gdansk, Poland

## Abstract

*Background*. Despite success of thoracic sympathectomy (ETS), there are patients that develop postoperatively intensive sweating of the trunk. The aim of the study was to present outcomes of three of those methods: removal of the clips, clipping of T6-9, and regional abdomino-lumbar iontophoresis (RALI). *Methods*. Out of the group of 229 patients treated with ETS, there were 9 that requested removal of the clips, 3 were treated with T6-9 video thoracoscopic block, and 5 were treated with RALI. The intensity of the side effect has been evaluated subjectively (with overall and localized perception of intensity of sweating) and objectively (with gravimetry). *Results*. The removal of the clips resulted in slow (about 12 months) diminishing of the intensity of sweating of the trunk; but the symptom did not disappear to the degree satisfactory for the patients. The T6-9 block resulted in partial and transient diminishing of the symptom. The iontophoresis resulted in very promising short-term results. *Conclusion*. Removal of the clips from the sympathetic trunk does not provide resolution of compensatory sweating in 1 year of observation. T6-9 block does not provide remedy for compensatory hyperhidrosis. Regional abdomino-lumbar iontophoresis seems to be very promising, but further research and followup are mandatory.

## 1. Introduction

 Despite unquestionable and spectacular success of sympathectomy, there is still a group of patients that developed postoperatively intensive sweating of the trunk, known as compensatory or reflex sweating. Though the theories behind the origin of this phenomenon are still unclear [1], the existence of this side effect of sympathectomy is considered one of the most important limitations of the surgical treatment of primary hyperhidrosis and facial blushing, and it does not seem to diminish with time [2, 3]. The pathogenetic concepts behind the compensatory sweating (CH) have been presented in the Guidelines of Brazilian Thoracic Surgery Society (BTSS) in 2008 [1]. Although the frequency of compensatory sweating is varying between the studies (from 0 to 95% of patients) [1–6], the opinion of the patients, available from different websites, seems to indicate that this problem is very important [6, 7]. The intense CH is diagnosed in 1–4% of patients after sympathectomy [1]. In our previous studies, the prevalence of compensatory sweating reached 100%, but most of the patients declared CH as mild or acceptable [8]. It was only 13 (5.4%) of the patients suffered from strong or intractable CH (intense CH according to BTSS). Out of this group, it was only 10 who decided to search for further treatment of CH [9]. The description of the phenomenon of compensatory sweating has been presented in BTSS Guidelines by Lyra et al. [1].

 Various methods have been proposed in order to provide decrease in the intensity of this side effect of the operation. The behavioral ones involve weight control, normothermic diet, proper selection of clothes, or avoiding countries with hot and humid climate, while medical include topical agents (aluminium chloride, botulin toxin [10], or oxybutynin treatment [1]). Some of them involve removal of the potentially therapeutic activity (removal of the clips used for sympathectomy), and offers concentrate on minimizing the intensity of compensatory sweating with local or generalized therapy.

 The aim of this study was to present feasibility and outcomes of 3 methods used for reduction of compensatory sweating in the department of general, endocrine, and transplant surgery.

## 2. Material and Methods

 Out of the group of 229 patients treated with video thoracoscopic sympathectomy for primary hyperhidrosis, there were 9 patients that were qualified for declamping of the sympathetic trunk, 3 in whom R6-9 block has been performed, and 5 in whom regional abdomino-lumbar ionthophoresis (RALI) was instituted. Only 10 patients within this group were previously treated in our department. The other 7 were 6 patients referred from another centers treating hyperhidrosis and one patient with trunk hyperhidrosis as a result of traumatic lesion in the area of lower part of cervical vertebral column. The median period from sympathectomy (or injury in 1 case) was for clip removal group 2.75 months (0.5–23), for R6-9 group 14 months (12–16), and for RALI group 14 months (12–24). The intensity of the side effect has been evaluated by gravimetric method described elsewhere [11, 12]. Apart from that a standard subjective method of evaluation was involved, with general sense of disease and particular intensity in different locations examined.

 Gravimetry is a short and easy method of objective measurement of sweating. It is performed by 1 min. collection of sweat with 3-weighted cotton swab. Net weight is calculated as the difference between brutto and tara weights. To include individual differences determined by the area in which the sweating is preferred, the net weight is divided by the total body surface and therefore standardized. Then reference values of sweating calculated on a sample of more than 400 healthy volunteers are presented in Table 1 [11].

 Thoracoscopic removal of the clips from the sympathetic trunk has been performed in all cases in general anesthesia with patient placed in prone position with arms abducted above head. The patients were intubated with standard tracheal tube and 8 mmHg carbon dioxide insufflation was used to obtain pneumothorax necessary for visualization of the thoracic sympathetic trunk. The previously placed clips were identified, gently dissected, and removed. Redon tube was then inserted, insufflation stopped, and vacuum suction instituted while the anesthetist was reinsuflating the lung with 5 to 10 manual breaths. Then the tube was removed and the wounds sutured. The procedure was repeated on the contralateral side.

 R6-9 sympathectomy was performed with the same positioning of the patient and the same procedure. The level of sympathectomy was determined by counting the ribs. Titanium clips were placed above and below sympathetic ganglia defined as T6, T7, T8, and T9. The desufflation on the thoracic cavity has been performed as described above. The procedures were also performed bilaterally.

 Regional abdomino-lumbar iontophoresis (RALI) was performed with the use of commercially available device for iontophoresis (Idromed 5DC, Bindner Medizin technik, Teningen, Germany) and gel electrodes (RFA High-Power Patient Return Electrode, Covidien, Poland). The selection of electrodes has been supported by a set of experiments and presented in another study. RALI included 21 initiating sessions for every area affected by sweating. Every 5 sessions direction of the current has been changed to opposite. The maximum intensity was 8 to 10 mA and was determined by the presence of burning and pain during session. In every such situation the patients were advised to increase the intensity of pain, and if any actual burn occurred, the area of burn was avoided in further sessions and treated with sulfathiazolum argentum cream. After the initiating sessions, the patients were performing sustaining sessions every 2-3 days.

 The intensity of sweating in primary and secondary localizations was evaluated prior to institution of the treatment and during follow-up visit 12 months afterwards.

 This study has been designed as a case-control, nonrandomized, comparison study due to the small sample and specificity of the problems that made patients search for medical help. The statistical analysis involved ANOVA and posthoc Scheffe tests and U Mann-Whitney comparisons. Every time *P* < 0.05 was considered significant. STATISTICA 9.0 PL software, licensed for Medical University of Gdansk, was used analysis.

## 3. Results

The surgical description of the cases is given in Table 2.

In general, both the procedures of removal of the clips and R6-9 sympathectomy were safe and did not result in any complications. There was no mortality. The lasting of intercostal pain has been similar to the results of our entire series presented elsewhere [8]. In 2 out of 5 patients treated with RALI small single (<0.5 cm in diameter) burns occurred. They were treated successfully with local administration of sulfathiazolum argentum cream, but treatment was relatively long (in both cases about 4 weeks) and the burns left visible scars.

 The results of declamping did not occur at once and were visible in about 12 months (47% reduction, chi-square *P* < 0.05). The results of R6-9 sympathectomy did not provide statistically significant decrease in compensatory sweating. RALI resulted in the most immediate and sustained reduction of compensatory sweating (43.5% reduction, Chi-square *P* < 0.05).

**Figure 1:**
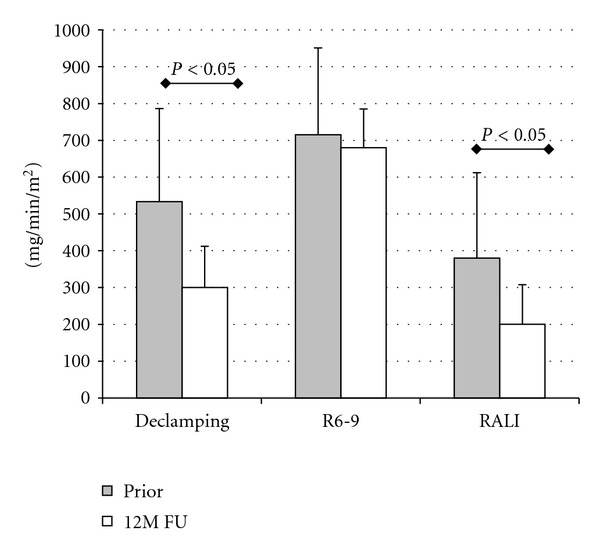
Objective (gravimetry) intensity of abdomino-lumbar sweating measure prior to intervention and 12 months after it. Comparisons in paired *t*-Student tests.

 The comparative objective abdominolumbar gravimetry results after 12 months are presented in Figure 1.

Subjectively, the most significant reduction of localized intensity of compensatory sweating was observed among patients treated with RALI (39.7% reduction, Chi-square *P* < 0.05). It is only this group of patients, who declared decrease in overall subjective intensity of disease (50% reduction, Chi-square *P* < 0.05). Interestingly, the patients that were treated with removal of the clips subjectively felt that both the intensity of their disease and subjective localized intensity od abdomino-lumbar sweating is higher than prior to the intervention (resp., 17.5% and 14.1% increase, Chi-square *P* < 0.05 and *P* < 0.1). Patients treated with R6-9 sympathectomy did not experience in a significant change in the overall subjective intensity of disease.

## 4. Comment

 In this study we presented preliminary results of 3 different methods potentially applicable to treatment of inacceptable compensatory sweating that results from previous sympathectomy for primary hyperhidrosis. We described the methodology of those 3 procedures, confirmed their safety and feasibility, and presented the long-term results. The highest degree of reduction of the symptoms was achieved with regional abdomino-lumbar iontophoresis (RALI), nevertheless, the objective evaluation of intensity of compensatory sweating indicated that the removal of the clips is associated with quite satisfactory results. Unfortunately, the specificity of the latter intervention involves the withdrawal of the primary therapeutic intention treatment of the primary hyperhidrosis. In that context, it is understandable that the patients treated with declamping declared increase in the overall sense of disease, as together with the decrease in compensatory sweating, they experience the recurrence of their initial suffering. Anatomically justified R6-9 sympathectomy did not turn to be effective. Neither objective (gravimetry) nor subjective results differed significantly from the assessments performed prior to intervention.

 It has been previously presented that clip removal results in 60–80% nerve recovery with 48–76% of patients recovering from intense CH with subsequent return to their initial symptomatology of PHH in at least 52% of patients [13, 14]. The remedy from intractable CH was achieved even in patients in whom the removal of clips was performed a year after the initial sympathetic block [13].

 In contrary, in a study on swine model, Loscertales et al. presented that the degree of degeneration of the sympathetic trunk after clipping is so high that there is practically no chance for nerve regeneration as early as 10 days after clipping [15].

 Alternative technique involves sympathetic chain reinnervation, either with sural nerve grafts or intercostal nerves. In a study by Telaranta, CH was significantly diminished after such an intervention in 81% of the patients, and 29% reported complete resolution of CH and return of primary hyperhidrosis [16]. Sympathetic chain reinnervation seems to be very promising, especially with the use of robotic surgery [17], but carries three disadvantages: demands sophisticated equipment, advanced skills and leads to the recurrence of the primary hyperhidrosis. It seems obvious that any form of more restrictive qualification protocol or locally or generally acting treatment of medication would offer much better therapeutic options than removal of clips of reinnervation with graft.

 It should be mentioned that each of the procedures evaluated in this study is associated with some specific disadvantages. Removal of all the clips, as presented above, does not result in immediate reduction of compensatory sweating and needs at least 6 months to produce any significant change. RALI does not provide full remedy from the unfavorable side effect of sympathectomy, is troublesome in application, demands repeated procedures, has to be stopped during pregnancy, and, in a significance frequency of cases, can lead to burns that heal slowly, and results in visible scarring. Nevertheless, this last method seems to be very promising as it helps to diminish the intensity of side effect of sympathectomy without removing its primary goal treatment of primary hyperhidrosis.

 It has to be underlined that the present study is only an initial presentation of a new concept of treatment of compensatory sweating and therefore carries several shortcomings. The samples are very small, there is no randomization, and the groups are only partially comparable in that initial intensity of the side effects. Thus it was mostly the extent of reduction of the problem that was the main end point of the study. For further and more reliable exploration of the subject more studies on larger groups are mandatory.

 There are further questions that still remain unanswered—for example when is the time limit for the removal of the clips or how the compensatory sweating changes over time and if it increases with subsequent lumbar sympathectomy. For those questions multicenter cooperation seems inevitable.

 In a study by Sugimura et al., the time for removal was from 1 to 57 months. 48% of the patients reported a substantial decrease in their compensatory sweating (5–10 on the visual analogue scale) after reversal. Interestingly, 42% reported that their initial hyperhidrosis or facial blushing has remained well-controlled (8–10 on the visual analogue scale) after reversal. The median follow-up period was 34 months. Based upon the period from sympathectomy to clip removal the authors divided the patients into early (up to 6 months) and late (more than 6 months). The differences did not reach statistical significance, but presented an interesting trend. In the early group, substantial decrease in CH was observed in 67% compared to 37% in the late group. Respectively, the postreversal satisfaction was excellent in 36% of the patients from early group compared to 22% of the late group. Finally, the control of primary disease (hyperhidrosis or facial blushing) was maintained in 25% of the early group and 53% of the late group [13].

 Not many studies have been undertaken to evaluate the influence of primary lumbar or sequence thoracic-lumbar sympathectomies on compensatory sweating [18–21]. It has been presented in a study on 56 patients submitted to thoracic followed by lumbar sympathectomy that 60.7% experienced increase of CH after the latter intervention [18]. In contrary, in a collaborative study by Rieger et al. the increase of compensatory sweating was observed only in 18% of the patients treated with lumbar sympathectomy following thoracic sympathectomy [21].

 In conclusion we would like to emphasize that both removal of the clips and regional abdomino-lumbar iontophoresis lead to objective reduction of compensatory sweating, but the latter procedure preserves the initial effect of sympathectomy and therefore maintains subjective benefits of the initial operation.

## Figures and Tables

**Table 1:** Reference values of sweating in different localizations evaluated with gravimetry [11].

Localization	Mean value in 1-minute test divided by body surface	Upper limit of acceptance defined as mean + 2 × standard deviation
Facial	16 mg/min/m^2^	55 mg/min/m^2^
Palmar	16 mg/min/m^2^	55 mg/min/m^2^
Axillary	40 mg/min/m^2^	125 mg/min/m^2^
Abdominolumbar	13.5 mg/min/m^2^	40 mg/min/m^2^

**Table 2:** Surgical characteristics of the operations.

Procedure	Removal of the clips	R6-9 sympathectomy
Time [mins]	25.5 ± 8.2	43.5 ± 14.1
Blood loss [mls]	0	25 ± 10
Opiod pain medication [hours]	12.5 ± 8	20.2 ± 5.2
Time to discharge	24 hours	24 hours (48 in one case due to prolonged intercostal pain)
Time to full recovery	7 days	14 days
Time to full remission of intercostal pain	5 days	32 days
